# Improved Esthetics Using Silver Diamine Fluoride in the Management of Early Childhood Caries: A Case Series

**DOI:** 10.7759/cureus.67275

**Published:** 2024-08-20

**Authors:** Mridula Goswami, Babita Jangra

**Affiliations:** 1 Pediatric and Preventive Dentistry, Maulana Azad Institute of Dental Sciences, New Delhi, IND

**Keywords:** staining, silver diamine fluoride, potassium iodide, pediatric dentistry, glass ionomer cement, dental caries

## Abstract

Dental caries prevention using an application of silver diamine fluoride (SDF) is an emerging advanced treatment modality in pediatric dentistry. However, a major drawback of SDF application is the black staining of carious lesions, which limits its clinical use due to esthetic concerns. Improving the esthetic outcome by reducing black staining would significantly enhance the opportunity for the universal use of SDF. This case series comprises three cases demonstrating the clinical success of using potassium iodide (KI) to reduce staining, followed by glass ionomer cement (GIC) restoration in primary molars. Upon 12-month follow-up visits, the treated teeth remained clinically asymptomatic, with esthetically acceptable restoration margins. Additionally, the esthetic results were acceptable to both parents and patients. The significance of utilizing KI and GIC following SDF application lies in their synergistic effect on masking discoloration resulting from SDF treatment while enhancing tooth functionality, thereby meeting patients' esthetic requirements and improving chewing efficiency.

## Introduction

Dental caries is a global health problem affecting a large population, despite advances in dental care over the past few decades [1]. The scenario in India is no different from that in other developed or developing countries with an overall prevalence of dental caries of 54.16% [2]. Like other developed nations, India is also advanced in dental care, including the use of minimally invasive dentistry techniques such as silver diamine fluoride (SDF) [3]. However, the burden of untreated carious lesions remains high, despite increased awareness and improved care, with a consistent trajectory over the last couple of decades [4]. Topical application of SDF solution has proven effective in arresting active carious lesions, especially in patients with special needs, uncooperative very young children, or medically compromised patients. The technique of SDF is considered a simple, non-invasive, and cost-effective treatment modality to arrest dental caries [5]. However, the post-treatment black discoloration of the arrested caries lesion is not esthetically pleasing and can be unacceptable to children and their families [6,7]. Thus, there is a need to explore alternative methods that can yield better esthetics while retaining the caries-arresting outcome of SDF [6]. 

The application of potassium iodide (KI) solution after SDF application has been shown to improve esthetic outcomes in many independent studies [7-12]. This result is due to a reaction with free silver ions on the SDF-treated surface forming silver iodide (AgI), which creates a white creamy reaction product [12]. A simplified chemical reaction between SDF (Ag(NH3)2F) and tooth mineral hydroxyapatite (HA) (Ca10 (PO4)6 (OH)2) is shown as follows [13].

 Ca10 (PO4)6 (OH)2 + Ag(NH3)2F → CaF2 + Ag3PO4 + NH4OH

CaF2 → Ca++ + 2F− 

Ca10 (PO4)6 (OH)2 + 2F− → Ca10 (PO4)6 F2 + 2OH−

The silver phosphate (Ag3 PO4) that precipitates on the tooth surface is insoluble. Silver phosphate is initially yellow when formed, but it quickly turns black when exposed to sunlight or reducing agents. The calcium fluoride (CaF2) formed provides a reservoir of fluoride for the formation of fluorapatite (Ca10 (PO4)6 F2), which is more resistant to acid attack than hydroxyapatite (Ca10(PO4)6 (OH)2) [14].

The chemical reaction of SDF treated with KI has been proposed as follows:

 Ag(NH3)2F(aq)+KI(aq)→AgI(s)+2NH3(g)+F- (aq)

When a saturated solution of KI interacts with SDF, a creamy white precipitate of silver iodide crystals forms on the tooth surface. This reaction prevents the formation of black precipitates by free silver ions from SDF, effectively preventing tooth discoloration [15]. However, the application of SDF can only arrest the progress of dental caries. The unfilled cavitation and lost tooth structure compromise chewing ability and dental plaque control in the affected area [16].

Using glass ionomer cement (GIC) restoration over SDF application results in a positive pulpal response, aids in the formation of secondary reparative dentin, restores function, and increases the resistance of cavity margins to secondary caries development [12]. It also masks the discoloration produced by SDF application and provides restoration of the lost dentine and enamel [17]. 

The advantage of using KI after SDF application is that it helps in reduction of black staining. The application of GIC, which also releases fluoride, improves esthetics along with increasing chewing efficiency and restorability of the tooth [12]. The available literature shows the in vitro masking ability of KI and GIC after SDF application. However, its correlation with clinical conditions is less explored in the literature [8,9,12,18]. Thus, the present case series hereby illustrates the in vivo results of the color masking ability of the combination of KI and GIC restoration, without affecting the caries-arresting activity of SDF in primary teeth.

## Case presentation

**Figure 1:**
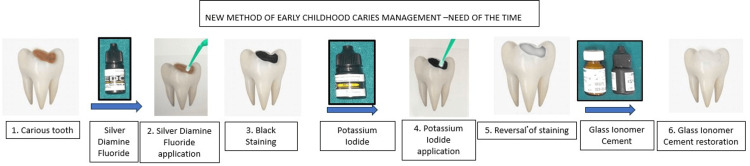
Diagrammatic representation of silver diamine fluoride application followed by potassium iodide and glass ionomer cement restoration in carious primary teeth Image credit: Babita Jangra

This case series features three unique cases in which SDF (Kedo SDF, Kedo Dental, India) and KI (Kedo SDF, Kedo Dental, India) application was followed by GIC (GC FUJI 9 Gold Label HS EXTRA GC, Japan) restoration in primary molars. All the cases presented to the Department of Pediatric and Preventive Dentistry with a chief complaint of carious teeth. The mean age of the children was six years. In each case, a detailed case history along with demographic details was recorded. The medical and dental history was non-contributory. The clinical examination was performed by a single calibrated and trained examiner with an assistant. Both clinical and radiographic records were made. Following informed consent from their parents and informed assent from the children, the necessary treatment was carried out in each case. In each case, SDF and KI application (according to the manufacturer’s instructions) was followed by GIC restoration of carious primary molars (Figure 1).

Clinical steps of SDF and KI application

Gross debris was removed from cavitation to ensure SDF reaches the carious tooth tissue or area of the tooth it is being applied to. Petroleum jelly was applied to the lips and gingiva to reduce the chance of temporary staining. The area was isolated with a cotton roll and the carious lesion or tooth tissue was dried with a gentle flow of compressed air or a cotton roll. One drop of SDF liquid (Kedo SDF, Kedo Dental, India) was dispensed in a disposable plastic dappen dish. SDF was applied using an applicator tip. SDF was applied and allowed to wait for 3-5 minutes. The excess solution was removed with moist cotton. KI (Kedo SDF, Kedo Dental, India) solution was applied immediately after SDF application using another applicator tip till discoloration was removed. The solution was removed with moist cotton.

Case 1

**Figure 2:**
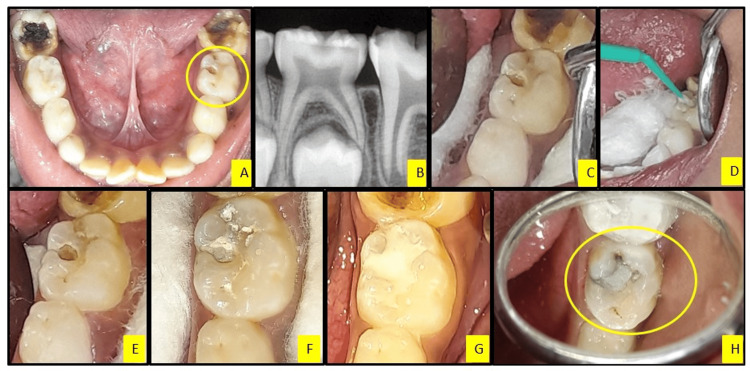
Silver diamine fluoride application followed by potassium iodide and glass ionomer cement restoration in the mandibular primary left second molar (75) A. Pre-operative mandibular occlusal view showing carious teeth 36,75,46, B. Radiographic view of the primary left second molar (75), C. Carious lesion after removal of gross debris, D.  Silver Diamine Fluoride application with a micro-applicator brush, E. Black staining of the carious lesion, F. After potassium iodide application, G. Immediate post-operative view after glass ionomer cement restoration, and H. 12-month follow-up

An eight-year-old male patient reported to the Department of Pediatric and Preventive Dentistry with a chief complaint of carious teeth in the lower left and right back tooth regions. Intra-oral examination revealed clinically decayed lower second primary molars and permanent first molars (75,36,85,46) (Figure 2A). The mandibular primary left second molar (75) was clinically and radiographically evaluated, and the treatment procedure was performed (Figures 2B-2G) as mentioned above. At the 12-month follow-up, the SDF and KI-treated tooth was clinically asymptomatic, with minimal staining of restoration margins, which was acceptable to the parent and patient (Figure 2H).

Case 2

**Figure 3:**
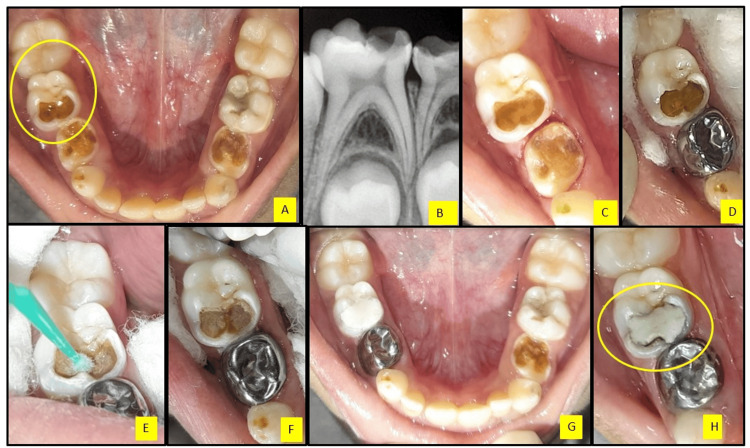
Silver diamine fluoride application followed by potassium iodide and glass ionomer cement restoration in the mandibular primary right second molar (85) A. Pre-operative mandibular occlusal view showing carious teeth 75,74,83,84,85, B. Radiographic view of the primary right second molar (85), C. Carious lesion after removal of gross debris, D. Black staining of the carious lesion after silver diamine fluoride application, E. Potassium iodide application with a micro-applicator brush, F. White precipitates after potassium iodide, G. Immediate post-operative view after glass ionomer cement restoration, and H. 12-month follow-up

A six-year-old male patient reported to the Department of Pediatric and Preventive Dentistry with a chief complaint of carious teeth in the lower left and right back tooth regions. Intra-oral examination revealed clinically decayed lower first and second primary molars (74,75,84,85) (Figure 3A). The mandibular primary right second molar (85) was clinically and radiographically evaluated, and a treatment procedure was performed (Figures 3B-3G) as mentioned above. At the 12-month follow-up, the SDF and KI-treated tooth was clinically asymptomatic with minimal staining of restoration margins, which was acceptable to the parent and patient (Figure 3H).

Case 3

**Figure 4:**
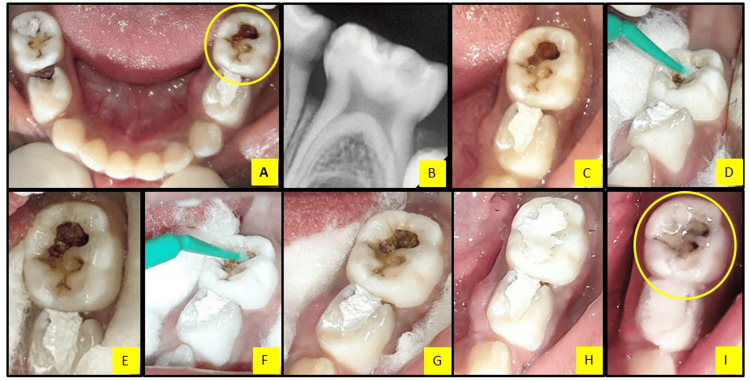
Silver diamine fluoride application followed by potassium iodide and glass ionomer cement restoration in the mandibular primary left second molar (75) A. Pre-operative mandibular occlusal view showing carious teeth 75,84, B. Radiographic view of the primary left second molar (75), C. Carious lesion after removal of gross debris, D. Silver diamine fluoride application with a micro-applicator brush, E. Black staining of the carious lesion after silver diamine fluoride application, F. potassium iodide application with a micro-applicator brush, G. White precipitates after potassium iodide, H. Immediate post-operative view after glass ionomer cement restoration, and I. 12-month follow-up

A five-year-old female patient reported to the Department of Pediatric and Preventive Dentistry with a chief complaint of carious teeth in the lower left and right back tooth regions. Intra-oral examination revealed a clinically decayed left second primary molar and right first primary molar (75,84) (Figure 4A). The mandibular primary left second molar (75) was clinically and radiographically evaluated, and the treatment procedure was performed (Figures 4B-4H) as mentioned above. At the 12-month follow-up, the SDF and KI-treated tooth was clinically asymptomatic with minimal staining of restoration margins, which was acceptable to the parent and patient (Figure 4I).

## Discussion

SDF’s safety and efficacy as a caries-arresting agent are well documented. However, there is a significant esthetic barrier that hinders its widespread acceptance among children. Recent trends have seen a paradigm shift in patient expectations regarding advanced dental care, with a high focus on esthetic outcomes [19]. Presently, the acceptance of color remains a critical factor when utilizing any preventive or restorative treatment modality [12]. Crystal et al. (2017) reported that one-third of parents rejected SDF treatment despite its benefits for dental caries in their child based on esthetic concerns [19].

Alshammari et al. (2019) reported that parents accepted SDF better in posterior teeth compared to anterior teeth in Saudi Arabia [20]. Various approaches are under study to reduce discoloration caused by SDF, and one such procedure gaining popularity is the application of KI [21]. Another method under consideration and trial is the use of a 20% glutathione (GSH) solution [22]. Goswami et al. (2024) reported the masking of discoloration with Bioflx crowns in primary molars after SDF application has better esthetic outcomes compared to chemical solutions [23].

Nevertheless, the application of KI over SDF has shown remarkable in vitro and in vivo results. In the present case series, SDF application was done on the carious lesions, followed by the immediate application of KI, and finally restored with GIC restoration. The potential masking of discoloration caused by SDF was achieved by the immediate application of KI (Figures 2F, 3F, 4G). Knight et al. (2005) proposed that using KI after the application of SDF utilizes all the remaining free silver ions on the surface to precipitate creamy white silver iodide (AgI) layers. Hence, free silver ions are no longer available to react with sulfur and other reagents in the mouth to form black precipitates in the teeth [24].

Kamble et al. (2021) reported that KI effectively reduced discoloration after the application of 38% SDF [25]. Patel et al. (2018) concluded that the use of KI immediately after SDF application resulted in no noticeable staining of the carious dentine or surrounding enamel [10]. Detsomboonrat et al. (2022) reported that KI application after SDF was able to reduce the degree of black staining in a dose-dependent manner, with minimal color change over a 14-day period [26]. Nguyen et al. (2017) also reported that KI showed minimal to no staining after four weeks of SDF application [8]. Gadallah et al. (2023) reported a color change assessment using a spectrophotometer, with the highest mean value recorded in the SDF group, followed by the SDF-KI group. The study also reported that there was no statistically significant difference in the percent change of surface microhardness with SDF-KI, despite the scavenging of silver ions [27].

In the present case series, SDF and KI-treated teeth were further restored with GIC, resulting in an acceptable restoration margin. The functionality of the tooth was restored with the GIC restorative material. This also contributed to significant plaque control. The high concentration of fluoride ions released by SDF and GIC together may help in arresting active carious lesions and prevent the development of new carious lesions in the treated teeth making it a good choice for use in primary teeth [14].

Knight and McIntyre (2006) compared the bond strengths of auto-cure glass ionomer cement to dentin surfaces that had been treated with AgF and KI, and those without treatment. The study reported that the application of AgF/KI to etched dentin samples, followed by washing off the precipitate, resulted in bond strengths that were not significantly different from conditioned samples [28]. Koizumi et al. (2006) reported that the adhesive strength of GIC and etch-and-rinse adhesive were less affected by the Riva Star (SDF and KI) application [29].

Zhao et al. (2017) also reported that SDF and KI treatment inhibited the development of secondary caries on GIC restorations. SDF and KI treatment caused perceptible staining at the restoration margin, but the intensity was much less than with SDF treatment alone [12]. Similar results were achieved in the present case series, with minimal discoloration of the restoration margins over the follow-up period. However, this marginal discoloration was esthetically acceptable to both the parents and the patients.

## Conclusions

SDF application on primary molars arrests the progression of carious lesions and further prevents the development of new carious lesions. However, the resultant black discoloration statistically decreases its acceptance amongst parents and children. Recent treatment modalities, such as the co-application of a KI solution after SDF treatment, significantly reduce post-treatment discoloration. Using a restorative material, such as GIC, over the treated tooth enhances the masking and restores the functionality of the tooth, while also contributing to future plaque control. This markedly improves the child’s quality of life, making it an effective sandwich method to provide caries protection with reasonable esthetic results.
